# Factor Structure of Early Smoking Experiences and Associations with Smoking Behavior: Valence or Sensitivity Model?

**DOI:** 10.3390/ijerph10126305

**Published:** 2013-11-26

**Authors:** Stéphanie Baggio, Joseph Studer, Stéphane Deline, Meichun Mohler-Kuo, Jean-Bernard Daeppen, Gerhard Gmel

**Affiliations:** 1 Alcohol Treatment Centre, Lausanne University Hospital CHUV, Av. Beaumont 21 bis, Pavillon 2, CH-1011 Lausanne, Switzerland; E-Mails: Joseph.Studer@chuv.ch (J.S.); Stephane.Deline@chuv.ch (S.D.); Jean-Bernard.Daeppen@chuv.ch (J.-B.D.); Gerhard.Gmel@chuv.ch (G.G.); 2 Institute of Social- and Preventive Medicine, University of Zurich, Hirschengraben 84, CH-8001 Zurich, Switzerland; E-Mail: meichun.mohler-kuo@uzh.ch; 3 Addiction Switzerland, postale 870, CH-1001 Lausanne, Switzerland; 4 Centre for Addiction and Mental Health, 250 College St., Toronto, ON, M5T 1R8, Canada; 5 University of the West of England, Frenchay Campus, Coldharbour Lane, Bristol BS16 1QY, UK

**Keywords:** early smoking experience, factor structure, smoking behavior, tobacco

## Abstract

The Early Smoking Experience (ESE) questionnaire is the most widely used questionnaire to assess initial subjective experiences of cigarette smoking. However, its factor structure is not clearly defined and can be perceived from two main standpoints: valence, or positive and negative experiences, and sensitivity to nicotine. This article explores the ESE’s factor structure and determines which standpoint was more relevant. It compares two groups of young Swiss men (German- and French-speaking). We examined baseline data on 3,368 tobacco users from a representative sample in the ongoing Cohort Study on Substance Use Risk Factors (C-SURF). ESE, continued tobacco use, weekly smoking and nicotine dependence were assessed. Exploratory structural equation modeling (ESEM) and structural equation modeling (SEM) were performed. ESEM clearly distinguished positive experiences from negative experiences, but negative experiences were divided in experiences related to dizziness and experiences related to irritations. SEM underlined the reinforcing effects of positive experiences, but also of experiences related to dizziness on nicotine dependence and weekly smoking. The best ESE structure for predictive accuracy of experiences on smoking behavior was a compromise between the valence and sensitivity standpoints, which showed clinical relevance.

## 1. Introduction

Initial subjective experiences of cigarette smoking play an important role in determining the likelihood of continued smoking and the development of nicotine dependence [1,2,3,4,5]. Indeed, most of the reinforcing effects of drugs are associated with positive initial subjective experiences [6], but most of the continuing smokers did not report pleasant experiences about their first tobacco use [2,7,8]. Thus, initial adverse experiences may be followed by tolerance and dependence [3,4,9,10]. These apparently contradictory results may be understood from two different perspectives, which are in some ways opposed. The current study examined these two standpoints and aimed to assess which one was best adapted to the Early Smoking Experiences (ESE); it also looked at some issues related to the measurement of initial subjective experiences. 

### 1.1. Perspectives on Initial Subjective Experiences of Tobacco

The first perspective examined was the valence model; this separates positive from negative experiences. In this model, positive initial experiences related to first smoking (relaxation, pleasurable effects) are predictive of continued use and nicotine dependence [4,9,11,12,13,14,15,16,17]; negative experiences (dizziness, nausea, irritation) discourage continued smoking [5,9,15,16,18,19]. 

The second perspective examined was the sensitivity model [3]; this suggests that the initial sensitivity to nicotine predicts tolerance and dependence [20]. This model suggests that smokers with a high innate sensitivity to nicotine mainly experience the two symptoms of dizziness and relaxation [4,21], and are more likely to progress to regular smoking [3,4,9,10,17]. From this perspective, other negative initial experiences do not necessarily prevent continued use and later dependence.

### 1.2. Methodological Issues and Measurement of Initial Subjective Experiences of Tobacco

An ideal method of assessing initial subjective experiences of tobacco would include physical and psychological measures just after first use [22]. As this is not feasible, retrospective questionnaires commonly ask participants to recall and rate their initial feelings [21]. We used the most widely used scale for assessing initial subjective experiences of tobacco: the Early Smoking Experience questionnaire (ESE, [15]). Previous studies reported its stronger reliability [17]. Nevertheless, three problems must be mentioned about how the ESE has been used in the literature so far. 

A total of eight items are used to assess participants’ impressions of their first few cigarettes (*i.e.*, pleasant sensations, unpleasant sensations, nausea, relaxation, a pleasurable rush or buzz, coughing, dizziness, and difficulty inhaling). The first problem is that most of studies relied on adapted versions of the initial ESE, for example, Riedel *et al.* [22] used five items (coughing, dizziness, feeling sick, feeling high, relaxation) and Okoli *et al.* [23] used seven items (dizziness, coughing, feeling sick, feeling high, relaxation, nervousness, feeling good). DiFranza *et al.* [4] proposed a more detailed questionnaire with 11 items related to irritation (coughing, chest pain, irritated eyes, bad taste in the mouth), nausea (upset stomach, feelings of wanting to vomit, vomiting), dizziness (feeling dizzy, lightheadedness, getting a rush or feeling a buzz), and relaxation (feeling relaxed). Rodriguez and Audrain-McGovern [16] proposed a validation of the ESE using the eight initial items, but then removed the “dizziness” item from the questionnaire because it loaded on both positive and negative factors. However, later studies added it again [5,17,21]. Thus, researchers have not been able to agree on precisely which items should be considered in the ESE.

A second problem occurs with the interpretation of the “dizziness” and “feeling a pleasurable rush or buzz” items on the ESE. As noted above, dizziness tends to load on both pleasant and unpleasant factors [5,16], or is described as a neutral item (neither positive nor negative [15]). This result does not fit the valence model, and some researchers deleted this item [16]. However, according to the sensitivity model, this item is important and should not be deleted, because it is highly predictive of continued use and dependence to tobacco. Likewise, defining and interpreting “feeling a pleasurable rush or buzz” is problematic; there is no reason to assume that a rush or a buzz is indeed a pleasant effect for all participants [21,24]. Dar *et al.* [25] showed that participants may feel that a “rush or buzz” is unpleasant and are influenced by the way sensations are defined in the questionnaire. O’Connor *et al.* [26] also reported that lightheadedness is “variously called dizziness, buzz, or high”, which are not equivalent in terms of positive or negative valence. Chen *et al.* [1] could not translate “rush or buzz” into Chinese, so they used “euphoria” instead; this is clearly not equivalent to “rush or buzz” and has positive connotations.

Finally, except for one study in China [1] and another in Hungary [17], the third problem is that the studies only took place in the United States and thus the ESE is under-studied in other cultures and contexts.

This study’s principal aim was to compare the two perspectives described (valence and sensitivity models) and to answer the methodological questions presented above within a German- and a French-speaking sample. For this purpose, the factor structure of the ESE was explored to examine how initial experiences were related to each other, and multiple-group comparisons were performed to test the invariance of the structure within the two groups. The effect of the different models on the prediction of smoking behavior was then tested, in order to know which one was best at predicting later use and dependence. 

## 2. Experimental Section

### 2.1. Participants and Procedures

The present study analyzed baseline data collected in the Cohort Study on Substance Use Risk Factors (C-SURF). C-SURF is a longitudinal study designed to assess substance use patterns and their related consequences in young Swiss men. Participants were enrolled between August 23rd 2010 and November 15th 2011 in three of Switzerland’s six army recruitment centers; these cover 21 of the country’s 26 cantons (including all French-speaking ones) and are located in Lausanne (French-speaking), Windisch and Mels (German-speaking). All young men around 20 years-old were eligible for study inclusion, because army recruitment is mandatory in Switzerland.

Of the 13,245 conscripts informed about the study, 7,563 (57.1%) gave written consent to participate; 5,990 of these (79.2%) filled in the baseline questionnaire. A total of 3,563 participants were lifetime tobacco users (59.5% of respondents). This study focused on the 3,368 participants who completed the whole ESE (94.5% of lifetime tobacco users) and missing values were listwise deleted. For the second part of the analysis, missing values on smoking behavior were also listwise deleted, leaving 3,216 participants (95.5% of the participants who completed the ESE). More information on sampling and non-response can be found in Studer *et al.* [27]. Briefly, non-respondents were more often substance users, but the differences between respondent’s and non-respondents were small. The study protocol (Protocol No. 15/07) was approved by the Ethics Committee for Clinical Research of Lausanne University Medical School.

### 2.2. Measures

#### 2.2.1. Initial Experience of Tobacco

The initial subjective experience of tobacco was assessed with an extended adaptation of the ESE [15], in line with DiFranza *et al.*’s version [4]. This version contained 10 items, including some that may be considered either positive or negative. These were pleasant experience, relaxation, felt not very well, felt dizzy/lightheaded, nausea, headache, stomach upset, heart pounding, coughed, eye irritations and bad taste in the mouth. The item which assessed rush or buzz was excluded from the questionnaire due to its redundancy with “felt dizzy/lightheaded”. Participants answered “yes” or “no” for each item.

#### 2.2.2. Smoking Behavior

Smoking behavior was assessed using 3 different measures. We assessed continued use of tobacco (had participants used tobacco in the past 12 months, “yes” or “no”), but also nicotine dependence (Fagerström Test for Nicotine Dependence, FTND, [28]), using a continuous total score ranging between 0 “very low” and 10 “very high”. We also calculated the number of cigarettes smoked weekly by multiplying the number of cigarettes smoked on a usual day and the number of days participants smoked per week. Nicotine dependence and cigarettes smoked were coded “0” for former smokers. Age of onset of tobacco use was also investigated.

#### 2.2.3. Socio-Demographic Variables

Socio-demographic covariates included age, language region (French- or German-speaking), perceived family income (participants were asked “*How well off is your family compared to other families in your country?*”. Answers were coded “below average income”, “average income”, “above average income”) and educational attainment (primary: 9 years of schooling; secondary: about 12 years; tertiary: 13 years or more, including university).

### 2.3. Data Analysis

For the purposes of this study we carried out two kinds of analysis.

#### 2.3.1. Exploratory Structural Equation Modeling (ESEM)

ESEM was performed to investigate the factor structure and to test the invariance of structure in the German- and French-speaking groups [29]. ESEM differs from traditional confirmatory factor analysis (CFA) in terms of factor loading estimations. Simple structures are tested by CFA (each indicator is influenced by only one factor) and rely on strong hypotheses about the factor structure; ESEM is less restrictive and allows loading matrix rotation and therefore loading variation across factors, as in exploratory factor analysis (EFA). ESEM provides more flexibility in a complex measurement structure. It also allows multiple-group EFA with measurement invariance, which is needed to test for structural invariance between the two language groups. ESEM was performed in four steps: (a) EFA were conducted within each linguistic group for ordinal data with unweighted least squares (ULS) estimation [30] in order to explore the structure and the number of factors of each group; (b) ESEM was performed for ordinal data with weighted least squares means and variance (WLSMV) adjusted estimation ([30] for both linguistic groups, allowing parameters to be free in each group (non-invariance model); (c) ESEM was then performed constraining all parameters to be equal for both groups (total invariance model) and Chi-square statistic between non-invariance model and total invariance model was computed to determine whether the structure was the same in each group; (d) a less restrictive model was used to test whether the same basic factor structure held for the two groups (configural invariance: same number of factors, same items salient to each factor across groups, but not necessarily exactly the same factor loadings), and a Chi-square statistic was also computed between non-invariance model and configural invariance model. Steps (b), (c) and (d) were performed twice, as two possible models were detected by step (a)’s EFA. Steps (a), (b) and (c) were summarized to put the emphasis on step (d) and the best model for ESE.

To assess the adequacy of the models, the following fit indices were used:

- The root mean square error of approximation (RMSEA), which is an approximation of the fit in the population. A value of 0.05 or less can be considered a good fit, whereas a RMSEA between 0.05 and 0.08 can be interpreted as an acceptable fit [31];
- The comparative fit index (CFI), which compares the result to the independence model. Values greater than 0.95 are considered a good fit, and those between 0.90 and 0.95 an acceptable fit [32];
- The standardized root mean square residual (SRMR) for EFA and the weighted root mean square residual (WRMR) for ESEM, which calculated the difference between observed and predicted correlations. Values under 0.10 for SRMR and 0.90 for WRMR are interpreted as good fits [33].

#### 2.3.2. Structural Equation Modeling (SEM)

SEM was then conducted on the different models to test their predictive accuracy effects on continued tobacco use, the number of cigarettes smoked weekly and nicotine dependence. As French- and German-speaking groups showed configural invariance in the previous analysis, both were included in the same SEM models, nevertheless controlling for language as there was minor variations in the ESEM. Both models considered the effects of two and three latent variables on continued use, nicotine dependence and the number of cigarettes smoked weekly. This time, we constrained the loading of experiences related to the other factor to zero in order to see the pure effect of each latent variable on smoking behavior. The fit indices described above were used to assess which model was best. SEM was performed controlling for socio-demographic variables (age, language, perceived family income, educational attainment and age of onset of tobacco use) and correlations between the 3 tested smoking behavior. The log of the variable for the number of cigarettes smoked weekly was used because of its important asymmetry. All the analyses were performed with Mplus 6.12 software [30].

## 3. Results and Discussion

### 3.1. Descriptive Results

Descriptive statistics and ESE frequencies are shown in Table 1. The more prevalent initial subjective experiences were items related to dizziness (did not feel very well, 38.8%; felt dizzy/lightheaded, 44.4%), positive experiences (liked the experience, 45.7%; felt relaxed, 44.2%) and irritation (coughed, 44.4%; felt irritation in the eyes and bad taste, 45.8%). Participants did not report many symptoms of dizziness (headache, nausea and upset stomach, heart pounding). Most of the participants continued to smoke (74.4%), but the number of cigarettes smoked weekly and nicotine dependence were quite low (39.6 cigarettes per week on average, mean FTND score = 1.5), even for continuing smokers (past 12 months smokers: 53.2 cigarettes per week on average, mean FTND score = 2.0).

**Table 1:** Descriptive statistics for initial experiences of tobacco and smoking behavior.

Variables			Overall N = 3,368	Language		Smoking status	
				(N = 3,368)		(N = 3,216)	
				French-speaking	German-speaking	Current smokers	Former smokers
				N = 1,745	N = 1,623	N = 2,392	N = 824
Initial experiences of tobacco ^1^							
	Did not feel very well		38.8	41.9	35.5	41.7	30.8
	Coughed		44.4	50.1	38.1	47.7	35.2
	Had a headache		22.4	30.7	13.6	23.2	20.1
	Felt irritation in the eyes and bad taste		45.8	48.9	42.4	48.2	38.2
	Had an upset stomach		5.8	5.6	6.1	6.4	3.6
	Felt heart pounding		14. 9	15.7	14.1	16.4	9.7
	Felt dizzy, lightheaded		44.4	40.1	49.0	47.2	36.8
	Felt nauseous		11.8	12.6	10.9	11.9	11.0
	Liked the experience		45.7	45.2	46.2	48.9	39.3
	Felt relaxed		44.2	38.3	50.6	48.5	32.0
Smoking behavior (N = 3,216)							
	Continued use (past 12 months) ^1^		-	78.0	70.5	100	0
	Nicotine dependence (FTND, score 0–10) ^2^		-	1.54 (2.04)	1.35 (1.98)	1.95 (2.12)	-
	Number of cigarettes smoked weekly ^2^		-	41.47 (54.26)	37.51 (51.99)	53.20 (55.52)	-
	Age of onset of tobacco use ^2^		-	14.62 (2.43)	14.50 (2.40)	14.43 (2.43)	-
Socio-demographic covariates							
	Age ^2^		20.30 (1.29)	20.36 (1.31)	19.66 (1.03)	20.06 (1.23)	19.91 (1.25)
	Education ^1^						
		Primary (9 years of schooling)	-	38.5	62.6	49.8	50.7
		Secondary (12 years of schooling)	-	29.3	24.3	27.8	24.4
		Tertiary (13 years of schooling or more)	-	32.1	13.2	22.4	24.9
	Perceived financial situation ^1^						
		Below average income	-	14.8	15.8	16.0	13.1
		Average income	-	48.4	30.7	40.2	39.2
		Above average income	-	36.8	53.4	43.8	47.7

^1^ Percentages are given; ^2^ Means and standard deviations are given.

### 3.2. ESE Factor Structure

**Figure 1:**
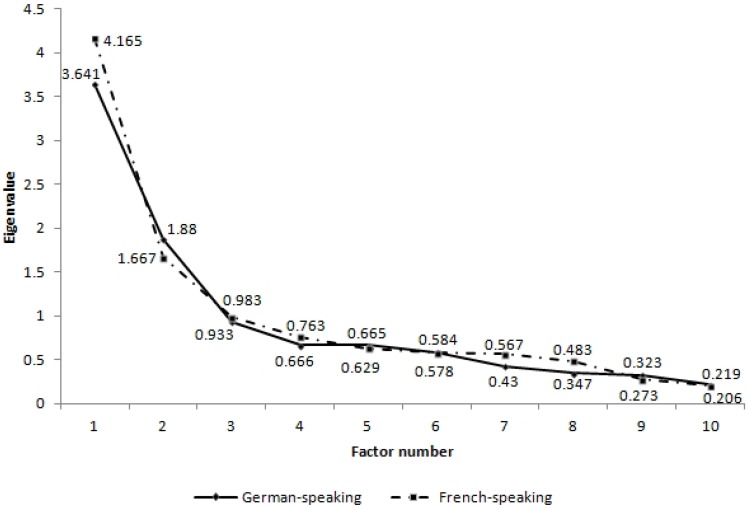
Scree-plot of eigenvalues for German-speaking EFA and French-speaking EFA.

(a) Initially, two EFA were performed on the 10 items of the ESE for the two groups separately. The eigenvalues showed that 2 or 3 factors could be selected for both of them, as shown on the scree-plot in Figure 1. According to Kaiser’s criteria [34], only the factors with eigenvalues equal to or higher than 1 should be selected; in both cases this was 2 factors. However, the third factors were close to 1 and explained almost 10% of the variance, so we tested the models using both two and three factors.

(b) The fit indices were a little better for the three-factor ESEM for non-invariance (two-factor model: RMSEA = 0.039, CFI = 0.977, WRMR = 1.478; three-factor model: RMSEA = 0.018, CFI = 0.997, WRMR = 0.765). Results indicated that the two-factor model included positive experiences on the first factor and negative experiences, whereas the three-factor model also included positive experiences, but separated negative experiences between irritation and dizziness (detailed results not shown, see following analysis for details about two- and three-factors models).

(c) There was no total-invariance across groups as the difference between these two models was significant for both the two-factor and three-factor models (two-factor model: *χ^2^*(2) = 139.001, *p* < 0.001; three-factor model: *χ^2^*(18) = 55.861, *p* < 0.001). Thus, the ESE factor structure was not exactly the same for French- and German-speaking participants.

(d) A less restrictive invariance was tested (same number of factors, same salient items for each factor). There was configural invariance for the three-factor model, but not for the two-factor model (two-factor model: *χ^2^*(2) = 6.921, *p* = 0.031; three-factor model: *χ^2^*(3) = 6.692, *p* < 0.082). The number of ESE factors and the items contributing to each factor were the same among French- and German-speaking participants (see Table 2). 

**Table 2:** Loadings for final ESEM three-factor model with configural invariance.

	German-speaking			French-speaking		
	(N = 1,542)			(N = 1,674)		
	1^st^ factor	2^nd^ factor	3^nd^ factor	1^st^ factor	2^nd^ factor	3^nd^ factor
**Did not feel very well**	0.258 ^**^	0.286 ^**^	−0.049	0.196 ^*^	0.668 ^***^	0.000
**Coughed**	0.015	0.664 ^***^	−0.024	0.771 ^***^	0.005	−0.081
**Had a headache**	0.473 ^***^	0.272 ^*^	0.002	0.178 ^*^	0.625 ^***^	0.061
**Felt irritation in the eyes and bad taste**	−0.018	0.654 ^***^	0.009	0.373 ^***^	0.273 ^***^	−0.018
**Had an upset stomach**	1.015 ^***^	−0.243 ^***^	0.001	0.001	0.779 ^***^	0.120 ^*^
**Felt heart pounding**	0.490 ^***^	0.060	0.174 ^**^	-0.047	0.610 ^***^	0.171 ^***^
**Felt dizzy, lightheaded**	0.240 ^*^	0.401 ^***^	0.246 ^***^	0.046	0.601 ^***^	0.250 ^***^
**Felt nauseous**	0.805 ^***^	0.001	−0.265 ^***^	-0.253	1.023 ^***^	−0.017
**Liked the experience**	−0.188	0.000	0.975 ^***^	0.001	-0.246 ^***^	0.857 ^***^
**Felt relaxed**	−0.040	0.008	0.735 ^***^	-0.040	0.008	0.735 ^***^

Remark: The last item (felt relaxed) was fixed across groups in order to identify the model. ^*^p < 0.05; ^**^p < 0.01; ^***^p < 0.001.

For French-speaking participants, the first factor was related to irritation (coughed, irritation in the eyes and bad taste), the second to dizziness (did not feel very well, headache, upset stomach, heart pounding, dizzy/lightheaded, nauseous) and the third to positive experiences (liked the experience, felt relaxed). For German-speaking participants, the first and second factors were inversed, but substantively the same. The first factor was related to dizziness and the second one to irritation (with cross-loading for “did not feel very well” and “felt dizzy/lightheaded”).

### 3.3. Predictive Accuracy of Initial Subjective Experiences on Smoking Behavior

**Figure 2:**
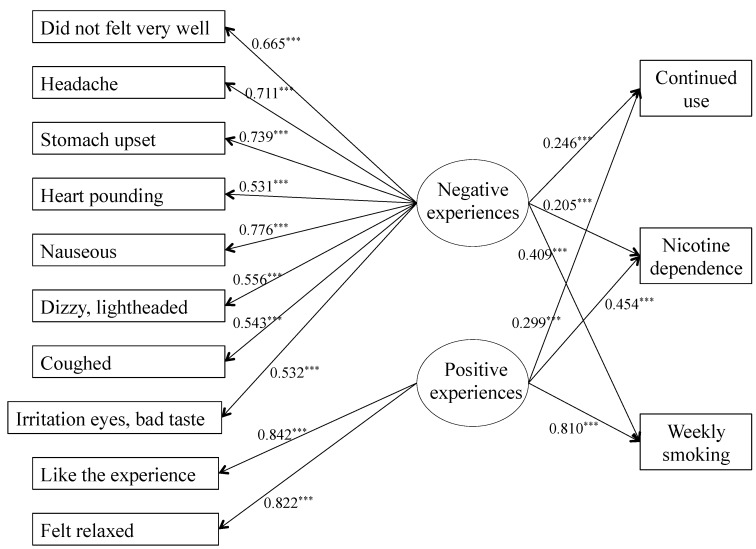
SEM for two-factor model.

**Figure 3:**
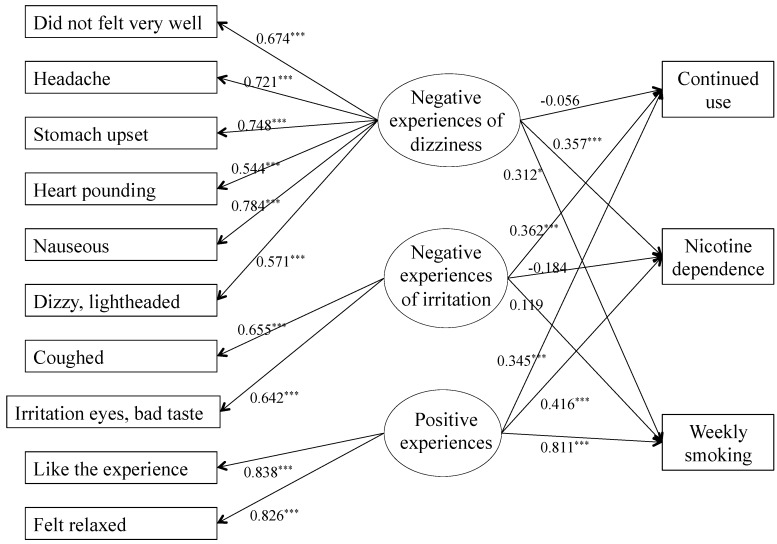
SEM for three-factor model.

The predictive accuracy on smoking behavior of the two models presented above were tested with SEMs and are presented in Figure 2 and Figure 3. In the two-factor model (Figure 2), both positive and negative experiences predicted continued use (negative experiences: *b* = 0.246, *p* < 0.001; positive experiences: *b* = 0.299, *p* < 0.001), nicotine dependence (negative experiences: *b* = 0.205, *p* < 0.001; positive experiences: *b* = 0.454, *p* < 0.001) and number of cigarettes smoked weekly (negative experiences: *b* = 0.409, *p* < 0.001; positive experiences: *b* = 0.810, *p* < 0.001). 

Fit indices: RMSEA = 0.042, CFI = 0.902, WRMR = 2.269. R^2^_continued use _= 0.156, R^2^_nicotine dependence_ = 0.138, R^2^_weekly number of cigarettes smoked_ = 0.154. ^***^p < 0.001. Model was tested controlling for age, language, perceived family income, education attainment and age of onset of tobacco use. Correlations between response variables were added: r_cotninued use, nicotine dependence_ = 0.835^***^, r_continued _use, weekly smoking = 0.818^***^, r _nicotine dependence, weekly smoking_ = 0.666 ^***^. For clarity, only pathways of interest are shown.

However, the three-factor model showed a difference between the two kinds of negative experiences (Figure 3). Only experiences related to irritation predicted continued use (*b* = 0.362, *p* < 0.001), whereas experiences related to dizziness predicted nicotine dependence (*b* = 0.357, *p* < 0.001) and number of cigarettes smoked weekly (*b* = 0.312, *p* < 0.001). Continued use was not predicted by dizziness (*b* = −0.056, *p* = 0.503), and irritation predicted neither nicotine dependence (*b* = −0.186, *p* = 0.111) nor the number of cigarettes smoked weekly (*b* = 0.119, *p* = 0.481). Positive experiences predicted continued use (*b* = 0.345, *p* < 0.001), nicotine dependence (*b* = 0.416, *p* < 0.001) and the number of cigarettes smoked weekly (*b* = 0.811, *p* < 0.001). The fit indices were a little better for the three-factor model (RMSEA = 0.041, CFI = 0.912, WRMR = 2.154) than for the two-factor model (RMSEA = 0.042, CFI = 0.902, WRMR = 2.269).

Fit indices: RMSEA = 0.041, CFI = 0.912, WRMR = 2.154. R^2^_continued use_ = 0.192, R^2^_nicotine dependence_ = 0.144, R^2^_weekly number of cigarettes smoked_ = 0.154. ^*^p < 0.05; ^***^p < 0.001. Model was tested controlling for age, language, perceived family income, education attainment and age of onset of tobacco use. Correlations between response variables were added: r_cotninued use, nicotine dependence_ = 0.855^***^, r_continued _use, weekly smoking = 0.817^***^, r _nicotine dependence, weekly smoking_ = 0.669^***^. For clarity, only pathways of interest are shown.

### 3.4. Discussion

This study considered the two main perspectives taken on initial subjective experiences of tobacco: a first one, which differentiates between positive and negative experiences, and a second one related to sensitivity to nicotine. According to the first perspective, positive and negative experiences may be distinguished from each other, and only the positive experiences should predict later use and dependence to tobacco. The second perspective underlines that symptoms of sensitivity to nicotine (dizziness, relaxation) should be predictive of the smoking behavior. This article aimed to explore the factor structure of the ESE and determine which perspective was more relevant, comparing two groups of German- and French-speaking Swiss young men.

Both the EFA and ESEM showed that two models could fit the results quite well. A two-factor model separated negative experience from positive ones, according to the valence model. A second, three-factor model, which showed a better fit, also had a factor related to positive experiences, but divided the negative factor between experiences related to dizziness and experiences related to irritations. Thus, the structure that best fitted the results was a compromise between the two perspectives: positive experiences formed a specific factor including both pleasant experience and relaxation (*i.e.*, an ESE item related to sensitivity to nicotine), but negative experiences were separated between dizziness (which is important in the sensitivity model) and irritation. No factor of sensitivity to nicotine could be found. This general structure fitted the German- and French-speaking groups equally well, however this was only configural invariance and the loadings differed from one group to another. The main loading difference was related to two experiences related to dizziness (“did not feel very well”, “felt dizzy/lightheaded”) which were loaded on the German-speaking group’s irritation and dizziness factors. However, dizziness did not load on the positive factor as was the case in some previous studies [15,16].

The predictive accuracy of these two models was then tested using SEMs, with the latent variables (positive and negative experiences for the first model; positive experiences, dizziness and irritations for the second) considered as predictors of continued use, nicotine dependence and number of cigarettes smoked weekly. Positive experience predicted all three outcomes in both models. This result was in accordance with previous studies: positive initial subjective experiences predicted later smoking behavior [4,13,15]. On the opposite side, a distinction in the effect of negative experiences appeared in both models’ results. In the two-factor model, negative experiences predicted continued use, nicotine dependence and the number of cigarettes smoked weekly. Negative experiences did not appear to be protective against later tobacco use. The three-factor model added a difference in the negative experiences’ effect on smoking behavior. Only the negative experience related to dizziness predicted nicotine dependence and the number of cigarettes smoked weekly. This result was relevant with the model of sensitivity to nicotine, because participants with high innate sensitivity to nicotine are more likely to be regular smokers and to develop dependence to nicotine [3,4,20]. On the other hand, experiences related to irritation predicted neither nicotine dependence nor the number of cigarettes smoked weekly.

However, the results were inversed when focusing on continued use. Experiences related to dizziness did not predict continued use, while experiences related to irritation encourage participants to continue smoking. This variable was perhaps less suitable for testing current smoking behavior than nicotine dependence or usual daily smoking, as it captured both regular and recreational users. Indeed, continued tobacco use may not be synonymous of an inability to quit smoking and nicotine dependence among the participants of this young cohort showed low levels of dependence (1.5 on a 0–10 scale) and use (39.6 cigarettes smoked per week on average). For this population, continued use may be synonymous of occasional smoking, which can be more influenced by the social context rather than personal affinity for tobacco use and sensitivity to nicotine, unlike nicotine dependence, which is a compulsive need to use tobacco including physical symptoms. To summarize, the three-factor model did not only seem to be the best model from a statistical point of view, but also from a clinical point of view, as it was best able to differentiate how subjective first experiences predict smoking behavior.

This study had some limitations—the main one being related to the true recollection of memories of first use. This recollection may be altered by continued use. Previous studies showed that current smokers had more positive recollections of their first tobacco use than former smokers [4,14,15]. However, our sample’s participants were quite young and the recall bias may be smaller in comparison to older smokers. Another limitation related to initial experiences with tobacco is the variability of exposure to nicotine among experimenters. Some of them may not have inhaled their first cigarettes deeply enough, and thus they had a reduced exposure to nicotine and its associated experiences and sensations [4,12]. A third limitation is the sample itself, composed exclusively of men. Participants were enrolled in Swiss army recruitment centers, but army recruitment is mandatory for men only in Switzerland. Thus, women did not represent a representative sample of young Swiss women, and enrolling women in such a context would have result in a biased sample. Therefore, our results should not be extrapolated to women. Further studies will have to consider gender differences in order to know whether results are the same for men and women.

## 4. Conclusions

In conclusion, this study showed that the best structure for the ESE was a compromise between the valence and the sensitivity models. Positive experiences were separated from negative experiences, but a distinction between experiences related to dizziness and to irritations appeared between the negative experiences. This pattern fitted both German- and French-speaking groups quite well and therefore seemed to be robust. Moreover, from a clinical point of view, experiences related to dizziness and to irritation had different predictive effects on smoking behavior. Thus these two kinds of experiences should not be grouped together and valence model should perhaps be dropped when studying the effects of initial subjective experiences on later smoking behavior.
